# MHC Variants Associated With Symptomatic *Versus* Asymptomatic SARS-CoV-2 Infection in Highly Exposed Individuals

**DOI:** 10.3389/fimmu.2021.742881

**Published:** 2021-09-28

**Authors:** Erick C. Castelli, Mateus V. de Castro, Michel S. Naslavsky, Marilia O. Scliar, Nayane S. B. Silva, Heloisa S. Andrade, Andreia S. Souza, Raphaela N. Pereira, Camila F. B. Castro, Celso T. Mendes-Junior, Diogo Meyer, Kelly Nunes, Larissa R. B. Matos, Monize V. R. Silva, Jaqueline Y. T. Wang, Joyce Esposito, Vivian R. Coria, Raul H. Bortolin, Mario H. Hirata, Jhosiene Y. Magawa, Edecio Cunha-Neto, Verônica Coelho, Keity S. Santos, Maria Lucia C. Marin, Jorge Kalil, Miguel Mitne-Neto, Rui M. B. Maciel, Maria Rita Passos-Bueno, Mayana Zatz

**Affiliations:** ^1^ Department of Pathology, School of Medicine, São Paulo State University (UNESP), Botucatu, Brazil; ^2^ Molecular Genetics and Bioinformatics Laboratory–Experimental Research Unit, School of Medicine, São Paulo State University (UNESP), Botucatu, Brazil; ^3^ Human Genome and Stem Cell Research Center, University of São Paulo, São Paulo, Brazil; ^4^ Department of Genetics and Evolutionary Biology, Biosciences Institute, University of São Paulo, São Paulo, Brazil; ^5^ Centro Universitário Sudoeste Paulista, Avaré, Brazil; ^6^ Departamento de Química, Faculdade de Filosofa, Ciências e Letras de Ribeirão Preto, Universidade de São Paulo, Ribeirão Preto, Brazil; ^7^ Department of Clinical and Toxicological Analyses, School of Pharmaceutical Sciences, University of São Paulo, São Paulo, Brazil; ^8^ Departamento de Clínica Médica, Disciplina de Alergia e Imunologia Clínica, Faculdade de Medicina da Universidade de São Paulo, São Paulo, Brazil; ^9^ Laboratório de Imunologia, Instituto do Coração (InCor), LIM19, Hospital das Clínicas da Faculdade de Medicina da Universidade de São Paulo, (HCFMUSP), São Paulo, Brazil; ^10^ Instituto de Investigação em Imunologia - Instituto Nacional de Ciências e Tecnologia-iii-INCT, São Paulo, Brazil; ^11^ Research and Development, Grupo Fleury, São Paulo, Brazil

**Keywords:** SARS-CoV-2, COVID-19, MHC, HLA, resistance, asymptomatic, MICA, MICB

## Abstract

Despite the high number of individuals infected by severe acute respiratory syndrome coronavirus 2 (SARS-CoV-2) who develop coronavirus disease 2019 (COVID-19) symptoms worldwide, many exposed individuals remain asymptomatic and/or uninfected and seronegative. This could be explained by a combination of environmental (exposure), immunological (previous infection), epigenetic, and genetic factors. Aiming to identify genetic factors involved in immune response in symptomatic COVID-19 as compared to asymptomatic exposed individuals, we analyzed 83 Brazilian couples where one individual was infected and symptomatic while the partner remained asymptomatic and serum-negative for at least 6 months despite sharing the same bedroom during the infection. We refer to these as “discordant couples”. We performed whole-exome sequencing followed by a state-of-the-art method to call genotypes and haplotypes across the highly polymorphic major histocompatibility complex (MHC) region. The discordant partners had comparable ages and genetic ancestry, but women were overrepresented (65%) in the asymptomatic group. In the antigen-presentation pathway, we observed an association between *HLA-DRB1* alleles encoding Lys at residue 71 (mostly DRB1*03:01 and DRB1*04:01) and DOB*01:02 with symptomatic infections and *HLA-A* alleles encoding 144Q/151R with asymptomatic seronegative women. Among the genes related to immune modulation, we detected variants in *MICA* and *MICB* associated with symptomatic infections. These variants are related to higher expression of soluble MICA and low expression of MICB. Thus, quantitative differences in these molecules that modulate natural killer (NK) activity could contribute to susceptibility to COVID-19 by downregulating NK cell cytotoxic activity in infected individuals but not in the asymptomatic partners.

## Introduction

Coronavirus disease 2019 (COVID-19), caused by severe acute respiratory syndrome coronavirus 2 (SARS-CoV-2) infection, became a worldwide pandemic affecting millions of people and the leading cause of death in Brazil in 2020 and 2021. Clinical manifestations range from severe cases with a lethal outcome to mild forms or asymptomatic cases. About 35%–50% of infected individuals are asymptomatic (1, 2) and are believed to be responsible for about 60% of transmissions (3).

Investigations on COVID-19 in thousands of samples from worldwide populations demonstrated the role of host genetics in disease susceptibility. Some variants and specific genome regions are related to disease severity and hospitalization (a proxy for disease severity), with different genome-wide association studies (GWASs) pointing to similar results. Among these associated regions and variants, we may cite rs11385942 at 3p21.31, in the region encompassing genes *SLC6A20*, *LZTFL1*, *CCR9*, *FYCO1*, *CXCR6*, and *XCR1*, rs657152 at 9q34.2 (the ABO blood group), rs10735079 at 12q24.13, in a gene cluster that encodes antiviral restriction enzyme activators (*OAS1*, *OAS2*, and *OAS3*), and rs74956615 at 19p13.2 (gene *TYK2*). There is also rs2109069 at 19p13.3 within the gene that encodes dipeptidyl peptidase 9 (*DPP9*) and rs2236757 at 21q22.1 in the interferon receptor gene *IFNAR2 (*4–7). One GWAS detected hits within the major histocompatibility complex (MHC), rs9380142 (*HLA-G*) and rs143334147 (*CCHCR1*) (5). However, while all these reported associations present p-values below the GWAS threshold (p < 10^-8^), the odds ratios (ORs) are very low (usually less than 1.5), and they cannot be considered predictive genomic markers of disease severity. Since many genes influence COVID-19 severity, polygenic risk must be considered. Major efforts have been made to evaluate polymorphism and disease severity, usually by comparing hospitalized patients with a population-based sample (the normal control), but they do not evaluate COVID-19 resistance in exposed individuals.

Identifying asymptomatic individuals or those resistant to the infection who are seronegative even after high exposure is challenging, since controlling for and measuring different degrees of exposure are complex. Asymptomatic or resistant individuals, however, may provide clues on the mechanisms of resistance and infection itself.

Genes modulating immune responses are natural candidates in studying resistance to infectious agents and disease outcomes. Together with other genomic regions, as the ones listed earlier, they may contribute to the “resistant’ phenotype. It has been shown that both innate and adaptive immune responses are crucial in the fight against COVID-19 (8, 9). In this context, the human MHC, harboring genes related to antigen processing, presentation, and immune modulation (10), is critical for both adaptive and innate immune responses. The human leukocyte antigen (HLA) genes within the MHC are among the most polymorphic genes in the human genome, and they are important targets for natural selection (11). The HLA class I molecules present antigens to CD8+ T lymphocytes (e.g., *HLA-A*, *HLA-B*, *and HLA-C*, usually expressed in all somatic cells) and HLA-class II to CD4+ T lymphocytes (e.g., *HLA-DRB1*, *HLA-DQA1* and *-DQB1*, *HLA-DPB1*, and others, usually expressed on professional antigen presentation cells, but also in activated T lymphocytes). In addition, HLA molecules play a critical role in the modulation of NK cell activity (e.g., *HLA-C*, *HLA-G*, *HLA-E*, and *HLA-F*, with a more restricted expression profile) (12). The MHC also harbors genes involved in antigen processing and loading (*HLA-DOA*, *HLA-DOB*, *HLA-DMA*, *HLA-DMB*, *TAP1*, and *TAP2*), cytokines such as tumor necrosis factor (TNF) and complement components, and genes that modulate the activity of NK cells, as *MICA* and *MICB*. The expression of *MICA and MICB* is low in normal tissues but is induced in tumors or during infections, upregulating (when expressed in the membrane) or downregulating (when expressed as soluble isoforms) NK cell cytotoxic activity (13).

Because of their unusually high polymorphism and extensive paralogy, GWAS findings for genes from the MHC are often ignored or treated with caution, as a consequence of HLA allele frequencies varied markedly across the world, and there may be different associations for different populations with diverse ethnicities. Because of that, the few studies on the frequency and distribution of HLA alleles and their clinical relevance for the SARS-CoV-2 infection (14–17) have shown conflicting results. However, the HLA locus is among the top hits in one GWAS from the COVID-19 Host Genetics Initiative (6).

To identify genetic factors involved as key players in the immune response of symptomatic SARS-CoV-2 infection and resistance, we have analyzed a cohort of couples discordant for the infection. While one was infected with symptomatic disease, the household-sharing partner, despite being closely exposed during the infection period, remained asymptomatic and seronegative for SARS-CoV-2 up to 6 months after the putative exposure. We whole-exome sequenced these couples, applied a bioinformatics pipeline to properly analyze variants within the MHC, and tested for genetic associations with disease/resistance phenotypes.

## Subjects and Methods

### Volunteers’ Recruitment and Datasets

Initial recruitment for screening involved broad media advertising based on the main inclusion criteria: couples discordant for SARS-CoV-2 symptomatic infection. From more than 2,000 received emails, we recruited 100 couples, all from São Paulo city (Brazil’s most populated metropolis). All couples filled out an online questionnaire, which included basic personal information (age, sex, blood type, comorbidities), and clinical progression of COVID-19 as well as diagnostic tests. The asymptomatic and seronegative member remained in close contact with his/her symptomatic partner throughout the SARS-CoV-2 infection, sharing the same bed (except when the symptomatic one needed to be hospitalized). Confirmatory tests (RT-PCR for symptomatic and RT-PCR or serology for asymptomatic) endorsed that just one of the pair had symptomatic viral infection at the time and that all asymptomatic are seronegative. The collection of biological samples occurred at intervals from 30 to 180 days after the reported viral infection. Serological testing was repeated in the blood plasma with two different techniques (electrochemiluminescence and ELISA-SARS-CoV-2 RBD/NP IgA and IgG). This excluded seven couples where the asymptomatic partner was found to have IgA or IgG antibodies against SARS-CoV-2. After exome sequencing quality control, we obtained data on 83 couples. To provide a baseline of allelic diversity and frequency, we also compared the exposed asymptomatic seronegative (the COVID-19[-] group) and the symptomatic group (the COVID-19[+] group) with MHC data from a previously whole-genome sequenced census-based sample of elderly individuals from the same city, pairing samples by gender and genetic ancestry (18). The characteristics of each group are available in **Supplementary Table S1**. Notably, the samples were collected between June and October 2020, before new SARS-CoV-2 variants were reported in Brazil (especially Gamma).

### Definition of Groups

The dataset is composed of couples sharing the same household and bedroom during the infection of one individual (**Supplementary Figure S1**). Three comparisons were conducted. Comparison 1 was made between COVID-19[+] *vs.* COVID-19[-] (n = 83 per group), with sex, age, and genetic ancestry as covariates. Due to the recurrent COVID-19 host hypothesis raised by our own analyses and literature about sex-specific factors driving infection risk, Comparison 2 subdivided the cohort into two sex-specific directions of resistance/susceptibility: COVID-19[+] males (n = 50) *vs.* COVID-19[-] females (n = 50) or COVID19[-] males (n = 28) *vs.* COVID-19[+] females (n = 28). In Comparison 2, homosexual couples were excluded of the analyses; age and ancestry were covariates. Lastly, Comparison 3 subdivided the cohort into two sex-specific groups: COVID-19[+] males (n = 51) *vs.* COVID-19[-] males (n = 29) or COVID-19[+] females (n = 32) *vs.* COVID-19[-] females (n = 54) using age and genetic ancestry as covariates.

### Exome Sequencing and Variant Calling

We used the Nextera Rapid Capture Custom Enrichment Kit or the Nextera Flex Kit (Illumina, San Diego, CA, USA) for library preparation and the IDT xgen-V1 kit for capture following manufacturer protocols. Whole-exome sequencing was performed on the NovaSeq 6000 equipment (Illumina, USA) with a 150-base paired-end dual index read format. Reads were aligned to the human reference GRCh38 using Burrow–Wheeler Aligner (BWA) (https://github.com/lh3/bwa/tree/master/bwakit). We also called genotypes using GATK HaplotypeCaller (version 4.0.9). The pipeline used for alignment, variant calling, variant refinement, and genetic ancestry assessment is detailed in the supplementary methods.

### Genotype and Haplotype Calls for the Major Histocompatibility Complex

**Figure 1:**
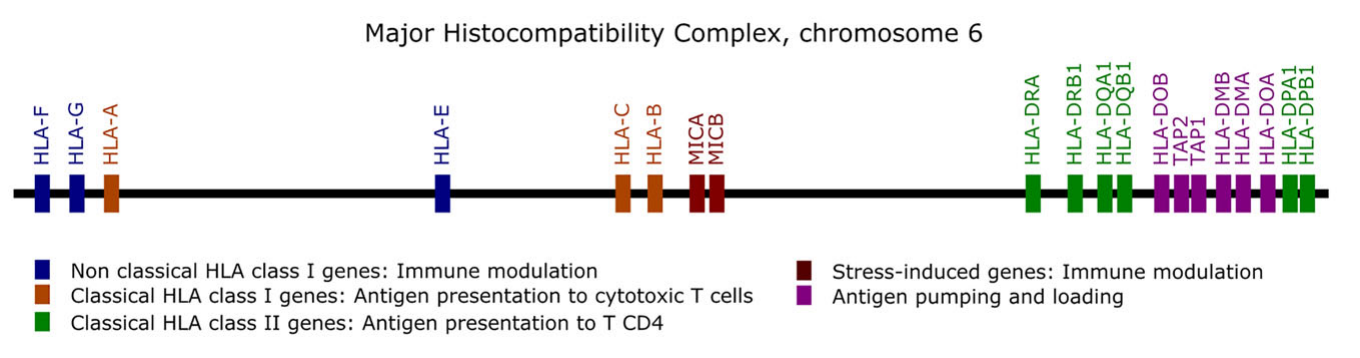
List of the genes optimized by the hla-mapper program in the Major Histocompatibility Complex (MHC).

Genes from the MHC are prone to alignment bias and genotyping errors because of the significant similarity and high polymorphism. To circumvent this issue, we used hla-mapper version 4 (19) to optimize read alignment for specific genes from the MHC (**Figure 1**), as described elsewhere (18, 19). After applying hla-mapper, we called genotypes using GATK HaplotypeCaller with a further refinement step using vcfx.

To obtain phased variants for each gene, we first phased close variants using GATK ReadBackedPhasing and then analyzed phase sets using the phasex program (https://github.com/erickcastelli/phasex), as described previously for a large Brazilian sample (18).

After the hla-mapper optimization and phasing, we obtained the complete exonic sequences for each individual and the translation of these sequences (**Supplementary Figure S2**). These methods are detailed in the supplementary methods, including the procedure to call HLA alleles.

### Statistical Analyses

Associations of phenotypic status with biallelic and multiallelic variants, allotypes, and specific amino acids were tested by fitting a logistic regression that considers each allele of a variant as an independent marker, controlling for age, genetic ancestry (all comparisons), and sex (when not stratifying by sex] using a local Perl script to convert the Variant Call Format (VCF) data into a plink-like table and R to fit the logistic regression. Due to sample size limitations, likely multifactorial inheritance, and the high number of variable sites (many multiallelic) in the MHC, we did not expect large effect sizes to be detected. Therefore, we used the threshold (p < 0.005) to detect candidates that may influence symptomatic infection susceptibility. To test if rare variants of larger effects contribute to the outcome, we also performed gene-based variant collapsing tests using SKAT-O within the rvtest program (20), enabling the analysis of multiallelic variants. SKAT-O is an optimized method for rare variants that combines and compares burden and SKAT tests, resulting in an optimal p-value for a given variant set (gene or gene set), controlling for the same covariates as individual variant associations.

## Results

We first compared the COVID-19[-] and COVID-19[+] groups, controlling for age, sex, and genetic ancestry (Comparison 1, **Supplementary Figure S2**). These groups had comparable ages, socioeconomic status, and genetic ancestry proportions (**Supplementary Table S1**). We observed a large sex difference between the two groups; 51 men and 32 women among symptomatic individuals compared to 29 males and 54 females among COVID-19[-]. Because some variants might be linked to symptomatic infection only in men or women, we also stratified the groups by sex (21) in comparisons 2 and 3 (**Supplementary Figure S2**). In all cases, for each group, we selected a population-based sample paired by sex and genetic ancestry to compare the frequencies with the expected in the general population.

**Figure 2:**
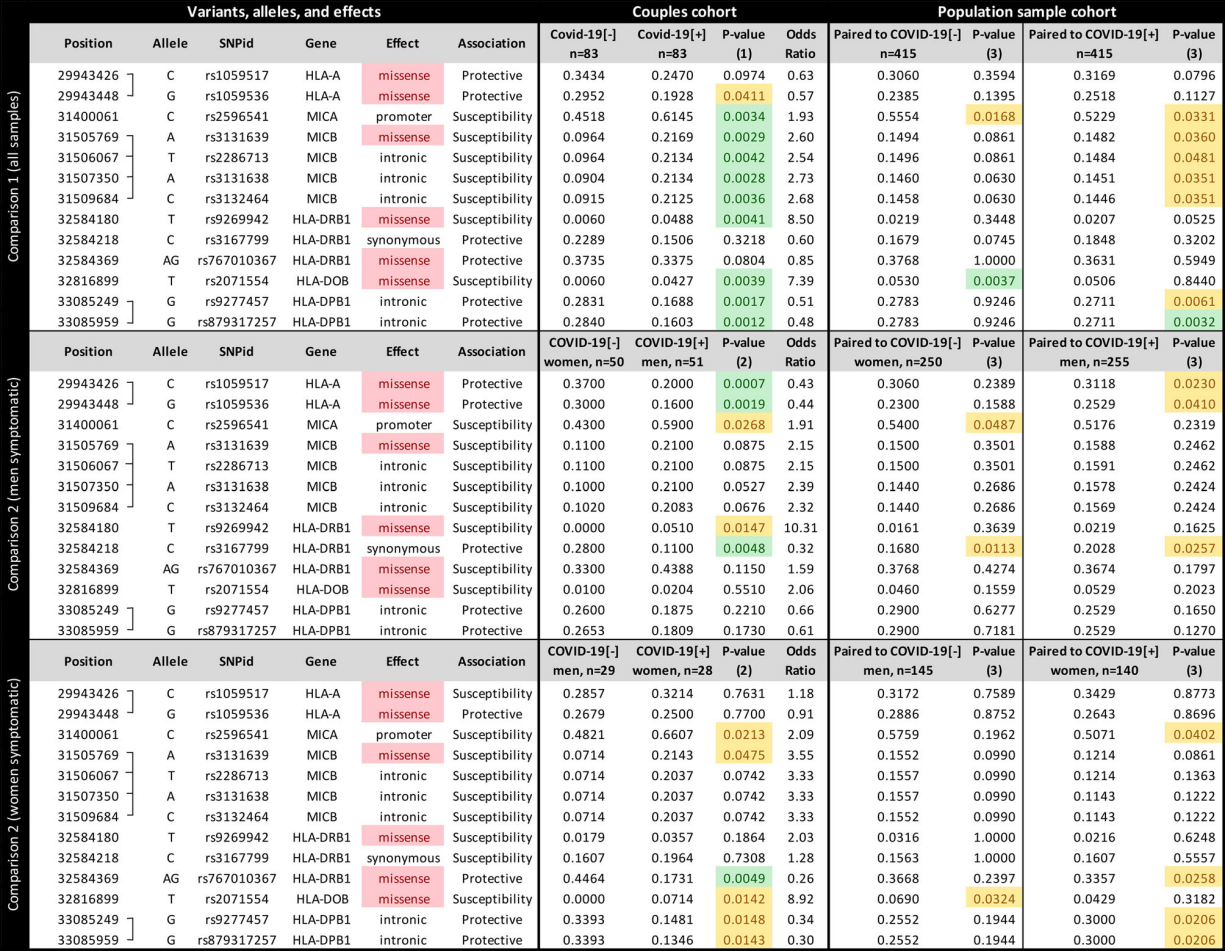
The frequency of each candidate variant at the Major Histocompatibility Complex (MHC) associated with susceptibility to SARS-CoV-2 symptomatic infection or with asymptomatic and seronegative after exposure. COVID-19[+]: Patients with symptomatic COVID-19. COVID-19[-]: Individuals sharing the same bed with symptomatic patients (exposed individuals) and are asymptomatic and seronegative. P-value (1): Logistic regression comparing all COVID-19[+] and all COVID-19[-] individuals, controlling for sex, age, and genetic ancestry; P-value (2): Logistic regression comparing COVID-19[+] and COVID-19[-] individuals, controlling for age, and genetic ancestry; P-value (3): Fisher exact test, comparing COVID-19[+] or COVID-19[-] individuals with a population sample paired by gender and genetic ancestry. In green, P-values < 0.005; In yellow, P-value between 0.005 and 0.05.

Among 1,723 queried variants within the genes illustrated in **Figure 1**, 13 attained significance at the p < 0.005 threshold (**Figure 2**). When controlling for sex, we found candidate variants for *MICA*, *MICB*, *HLA-DRB1*, *HLA-DOB*, and *HLA-DPB1* genes (**Figure 2** upper panel), including three missense variants in *MICB*, *HLA-DOB*, and *HLA-DRB1*. All variants from *MICB* and *HLA-DPB1* are in absolute Linkage Disequilibrium (LD). MICA promoter allele rs2596541/C is underrepresented in COVID-19[-] individuals when compared to symptomatic patients (p = 0.0034, OR = 1.9) and to the paired population sample (p = 0.0168) and overrepresented in the COVID-19[+] when compared to its paired population sample (p = 0.0331). Likewise, variants from *MICB* listed in **Figure 2** are overrepresented in COVID-19[+] compared with COVID-19[-] subjects (p < 0.005, OR = 2.6) and with the paired population sample (p < 0.05). The *HLA-DOB* missense variant rs2071554/T is underrepresented in the COVID-19[-] compared to COVID-19[+] (p = 0.0039, OR = 7.3) and the general population (p = 0.0037).

We detected different associations when stratifying the groups by sex. When evaluating sex-specific direction of infection within couples (comparison 2, **Figure 2** middle and lower panels), COVID-19[+] men and COVID-19[-] women, the strongest association is for two missense mutations in *HLA-A*, and this association is not detected when controlling for sex (**Figure 2** upper panel) or when testing for other directions (**Figure 2** lower panel). We also observed little to no effect of other MHC genes, except for a synonymous mutation in *HLA-DRB1* (rs3167799). *HLA-A* variants rs1059536/G and rs1059517/C, which are in high LD, are overrepresented in COVID-19[-] women compared to COVID-19[+] men (p < 0.005 and OR < 0.44 for both) and to the general population (p < 0.05). For COVID-19[+] women and COVID-19[-] men, the strongest association is for a missense mutation in *HLA-DRB1* (rs767010367), which is not detected in other comparisons. *HLA-DRB1* variant rs767010367/AG (the absence of a guanine deletion at chr6:32584370) is underrepresented in symptomatic women (p < 0.005, OR = 0.25) and in the general population (p < 0.05). When evaluating COVID-19[+] and COVID-19[-] individuals from the same sex (**Supplementary Figure S3**), we observed that the susceptibility *MICB* variants are overrepresented in symptomatic men and women, but significant only among men (p = 0.0128). The same can be observed for variants regarding HLA-DPB1. The associations regarding *HLA-A* are only observed when we compare COVID-19[+] men and COVID-19[-] women, but not when comparing individuals of the same sex. Finally, variant rs2071554 from HLA-DOB seems to be overrepresented only among COVID-19[+] women.

We applied SKAT-O in each candidate gene to evaluate the contribution of rare and common variants collapsed into gene-wide sets. Among MHC genes, we identified a significant association between *MICB* and COVID-19[+] (p = 0.017). We verified that the association signal was driven by the same common variants identified by the single variant association test described in **Figure 2**.

Most of the candidate variants in **Figure 2** presented intermediate frequencies in the general population samples when compared to the COVID-19[-] and COVID-19[+] groups, despite their similar genetic ancestry backgrounds. This finding may be explained by the mix of individuals prone to both phenotypes in the general population.

**Figure 3:**
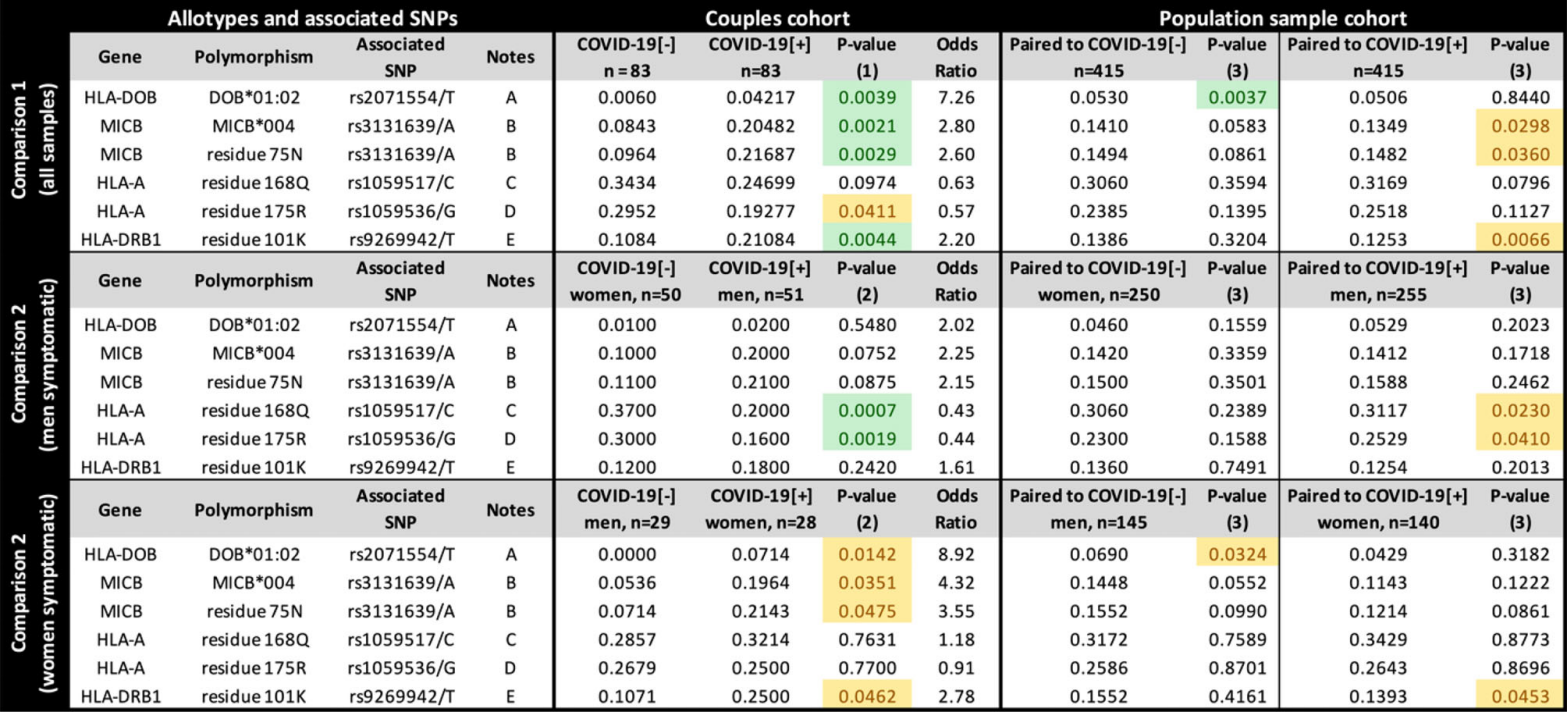
The frequency of each candidate allotype and amino acid residue encoded in the Major Histocompatibility Complex (MHC) associated with susceptibility to SARS-CoV-2 symptomatic infection or with asymptomatic and seronegative after exposure. COVID-19[+]: Patients with symptomatic COVID-19. COVID-19[-]: Individuals sharing the same bed with symptomatic patients (exposed individuals) and are asymptomatic and seronegative. P-value (1): Logistic regression comparing all COVID-19[+] and all COVID-19[-] individuals, controlling for sex, age, and genetic ancestry; P-value (2): Logistic regression comparing COVID-19[+] and COVID-19[-] individuals, controlling for age, and genetic ancestry; P-value (3): Fisher exact test, comparing COVID-19[+] or COVID-19[-] individuals with a population sample paired by gender and genetic ancestry. In green, P-values < 0.005; In yellow, P-value between 0.005 and 0.05. Notes: (A) DOB*01:02 is the only HLA-DOB allotype carrying residue 18Q; (B) MICB*004, *024, *028 carry residue 75N (residue 75 in the full-length protein); (C) This residue is common to many HLA-A alleles, including A*23:01, A*25:01, A*26:01, *29:02, *30:01, *30:02, *31:01, *32:01, *33:01; (D) This residue is common to many HLA-A allotypes, including A*23:01, *29:02, *30:01, *30:02, *31:01, *32:01, *33:01; (E) This residue occurs in DRB1*03:01, *03:02, *04:01,*04:09, *13:03. rs9269942/T captures only a fraction of the sequences encoding K at position 101 from HLA-DRB1, since the composition of other surrounding variants, including indels, can produce the codon for K.

To investigate how specific protein sequences or amino acid residues may influence susceptibility to SARS-CoV-2 symptomatic infection, we translated all the exonic sequences to proteins, *in silico*, comparing the frequency of every full-length protein (the allotypes) and every amino acid residue between groups (**Figure 3**). Considering all samples (**Figure 3**, upper panel), the allotype MICB*004 and the amino acid residue that defines it, 75-N, both related to the missense mutation rs3131639/A, and the presence of K at residue 101 from HLA-DRB1 (related to rs9296942/T), are overrepresented among COVID-19[+] when compared to COVID-19[-] (p < 0.005) and the general population (p < 0.05). Conversely, allotype HLA-DOB*01:02 and the residue that defines it, 18-Q, both related to the missense mutation rs2071554/T, are underrepresented in the COVID-19[-] when compared to their paired COVID-19[+] and the general population (p < 0.005).

When stratifying the groups by sex, the HLA-A residues 168Q and 175R, both corresponding to *HLA-A* missense mutations listed in **Figure 2**, are underrepresented in symptomatic men (p < 0.005) compared to COVID-19[-] women and in the general population (p < 0.05). However, we do not observe these differences when comparing COVID-19[+] and COVID-19[-] with the same sex (**Supplementary Figure S4**). HLA-DOB*01:02 is overrepresented mostly in COVID-19[+] women (p = 0.0044, OR = 9.0678). Overall, the protein and amino acid analyses corroborate what was observed in the single-nucleotide polymorphism (SNP) analysis for missense mutations.

We also tested *HLA-A* and *HLA-B* alleles grouped as supertypes (22). There is a trend to lower frequency of supertype A03 among exposed seronegative (p = 0.0378) when controlling for genetic ancestry, sex, and age, but this association was not significant after correction for multiple tests (six different HLA-A supertypes) or when stratifying samples by sex (**Supplementary Table S2**).

Finally, we discussed potential mechanisms underlying the associations presented in **Figures 2**, **3** (detailed at the **Supplementary Results**). In brief, the promoter variant from *MICA* is associated with higher mRNA expression levels (**Supplementary Figure S5**). In comparison, the susceptibility variants for *MICB* are associated with lower mRNA expression (**Supplementary Figure S6**). The susceptibility variant from *HLA-DOB* modifies the signal peptide and possibly the cellular localization and trafficking of the protein. The female *HLA-A* protective variants do not influence *HLA-A* expression levels.

## Discussion

Environmental factors such as protective measures, socioeconomic status, and access to healthcare may explain in part the high variability in COVID-19 disease incidence and mortality among individuals. However, few studies have focused on the genetics of resistance against SARS-CoV-2 infection due to limitations in controlling for exposure. Previous reports on host genetic factors with resistance to COVID-19 have investigated SARS-CoV-2 receptors such as the angiotensin-converting enzyme 2 (ACE2), the transmembrane protease serine-type 2 (TMPRSS2), glucose-regulated protein 78 kDa (GRP78), and the extracellular matrix metalloproteinase inducer or cluster of differentiation 147 (CD147) [reviewed by (23)].

GWASs addressing COVID-19 severity detected many different genomic regions and specific variants influencing the disease outcome (4–7, 24). However, it became clear that polygenic risk must be considered, since the ORs of these associations are relatively underpowered, usually lower than 1.5. The Brazilian media reported that both sibs from seven pairs of monozygotic adult twins died from SARS-CoV-2 infection within a few days of difference. Furthermore, recent observations showed that secondary transmission among close household contacts was 53% (25). These observations support the influence of host genetics in COVID-19 severity and resistance (6), particularly in a polygenic fashion.

An efficient response in the early course of SARS-CoV-2 infection may strongly influence infection outcome, differentiating CODIV-19-unaffected resistant individuals or asymptomatic after exposure and symptomatic ones. Several studies aimed to understand the human genetics of protective immunity to SARS-CoV-2 (24, 26, 27). Innate immune genes and genes involved in antigen presentation seem to be strong candidates in this matter, contributing with an additional layer to other known genomic regions that influence COVID-19 outcome (4–7, 24).

Spouses of infected and symptomatic COVID-19 patients (COVID-19[+]) sharing the same bedroom without protective measures represent an efficient approach to identify and ascertain resistant individuals highly exposed to the same viral strain of SARS-CoV-2. Here, we investigated 83 discordant couples, in which one was symptomatic and the partner remained asymptomatic and seronegative for at least 6 months. Since we collected all samples in the first semester of 2020, all couples were likely exposed to the same or closely related viral strains (28). Our study suggests that genes of innate and adaptive immune responses may play a vital role in susceptibility/protection to symptomatic SARS-CoV-2 infection. The innate response is key for controlling primary encounters with a pathogen (29). In the case of COVID-19, although SARS-CoV-2 is a novel pathogen, it shares extensive CD4+ and CD8+ T-cell cross reactivity with human endemic coronaviruses (30–32) and would therefore elicit a secondary T-cell response to the cross-reactive epitopes. This cross-reactive secondary T-cell response could eradicate SARS-CoV-2 infection in a proportion of individuals, even before a specific SARS-CoV-2 antibody response is made, as demonstrated in family contacts of COVID-19 patients (33, 34).

### Associations of the Antigen-Presentation Pathway

MHC variants showed no important associations between class I antigen-presentation genes (*HLA-A*, *HLA-B*, and *HLA-C*) and nonclassical HLA class I genes (*HLA-G*, *HLA-E*, and *HLA-F*) in symptomatic infection susceptibility. The only exception is two missense mutations of *HLA-A* protecting mainly women when the male partner is symptomatic (rs1059517/C and rs1059536/G), both related to some common allotypes such as HLA-A*23:01, *29:02, *30:01, *30:02, *31:01, *32:01, and A*33:01 (**Figure 3**). None of these alleles present relevant frequency differences when considered alone, but most of them are overrepresented in COVID-19[-] women, particularly HLA-A*23:01 and *29:02. These SNPs lead to two amino acid exchanges in the HLA-A molecule, encoding Q and R at residues 168 and 175 of the full protein (or 144 and 151 in the mature molecule), respectively. These residues belong to the alpha-2 domain, but they seem to not influence peptide binding (35), although residue 175 can interact with the T-cell receptor. The binding prediction of SARS-CoV-2 peptides to HLA class I genes indicated that alleles A*23:01 and A*29:02 are intermediate binders, thus enhanced antigen presentation should not be the case (36). Moreover, these variants seem to not influence *HLA-A* mRNA expression levels in men, women, or the combined sample. HLA-A*23:01 was detected as risk markers for severe COVID-19 among Spanish patients (37), but not in Russia (38). A GWAS detected a trend to a higher frequency of A*23:01 among the general population than in severe COVID-19 Italian patients, but no differences for A*29:02 (4). A study from China comparing COVID-19 patients and the general population did not detect any *HLA-A* association (39). Moreover, in our study, polymorphisms of nonclassical HLA genes involved in immune modulation (e.g., *HLA-E*) were not associated with symptomatic infection susceptibility in discordant couples, but a higher frequency of HLA-E*01:01 has been reported to be associated with hospitalized patients (9). In addition, we cannot rule out the influence of *HLA-G*, particularly variant rs9380142 as described in a recent GWAS (5), because this is a 3'-untranslated region (UTR) variant not captured by our exome analysis. Previous studies focusing on HLA genes and SARS-CoV-2 infection, comparing infection outcome (mild *vs.* severe COVID-19) or infection susceptibility in infected individuals against population control samples, show different associated alleles for each population and sample (4, 9, 14, 17, 38, 40). This lack of consistency among studies possibly reflects the wide frequency differences of HLA alleles among populations and how each study characterized its samples.

For HLA class II genes, we found an association of K at residue 101 of HLA-DRB1 when considering the full-length molecule (or residue 71 in the mature protein) with symptomatic infection susceptibility, mostly related to increased frequencies of DRB1*03:01 and DRB1*04:01 among symptomatic patients (**Figure 3**). HLA-DR molecules are important antigen-presenting molecules, essentially for CD4+ T cells, and lower HLA-DRB1 expression has been associated with the severity of the SARS-CoV-2 infection (41). Thus, inappropriate antigen-presentation by some HLA-DR molecules might facilitate infection susceptibility. Accordingly, DRB1*04:01 has been associated with lower HLA-DRB1 mRNA expression than many other alleles (42). Furthermore, these results conflict with previous data from other populations. For instance, in North East of England (43) and Saudi Arabia (44), HLA-DRB1*04:01 was 2–3 times more frequent in the asymptomatic group. Likewise, DRB1*03:01 had a protective effect against SARS-CoV-2 infection in the Sardinian population (40). In our survey, HLA-DRB1*04:01 and DRB1*13:03 were six times and three times more frequent among symptomatic individuals (p = 0.0187 and p = 0.0229, respectively), and DRB1*03:01 was also more frequent among symptomatic individuals (individually not significant). Taken together, they contribute to the much higher frequency of residue 71K among symptomatic individuals than exposed seronegative ones (p = 0.0044), which was also confirmed by the comparison with the general population (p = 0.0066). DRB1*04:01 is common in European populations (France, Denmark, England, Germany) and relatively rare in Asian and African populations (www.allele-frequencies.net). Conversely, DRB1*03:01 is common in Europe and Africa but uncommon in East Asia. In our sample, Brazilians carrying DRB1*04:01 present a much higher East Asian genetic ancestry (10.7%) than observed in the entire dataset (around 1.5%), while Brazilians carrying DRB1*03:01 present a similar ancestry background than the entire dataset. This lack of consistency for HLA-DRB1 associations among populations is another example of population-specific associations.

The DOB*01:02 (rs2071554/T, a missense variation in the HLA-DOB signal peptide) may have a potential effect in protein function, since *in silico* analysis of functional effects of this variation predicted a possible damage to the protein function (45). HLA-DO molecule is a modulator of HLA-DM, a peptide exchange factor required for efficient loading of endosomal peptides onto MHC-II molecules (46). This may lead to inadequate antigen presentation failure to recognize important epitopes.

### Natural Killer Cell Activity Pathway

Interestingly, most of the MHC class I region hits coincide with two genes, *MICA* and *MICB*. *MICA* and *MICB* are constitutively expressed in a few cell types, such as fibroblasts and epithelial cells but are markedly upregulated in stress conditions like cancer and infections (47). Here, the regulatory *MICA* variant associated with symptomatic infection is related to higher mRNA expression, possibly higher soluble MICA (sMICA), while the opposite is observed for all *MICB* variants (supplementary results). MICA and MICB interact with activating receptor C-type lectin-like receptor NKG2D and play an important role in mediating NK and TCD8+ cytotoxic activity (48).

Previous studies suggest the participation of the unconventional T (uT) cells, gamma-delta T (γδ T), NKT, and NK cells in the immune response against several infections such as tuberculosis, HIV, Influenza A, Influenza A (H1N1) [reviewed by (49)], as well as SARS-CoV-1 (50) and SARS-CoV-2 infections (51). These cells recognize non-peptide antigens in an MHC-independent way and produce mostly inflammatory cytokines such as interferon (IFN)-γ and eliminate target cells by perforin/granzyme-mediated cytotoxicity *in vivo (*52). Thus, these cells could be a key for a rapid defense against bacterial and viral infections (53) and contribute to control viral load through a higher MICB expression (from the exposed seronegative individuals), activating an effective natural response *via* NKG2D.

The missense *MICB* variant associated with SARS-CoV-2 symptomatic infection, MICB*004 and rs3131639/A, is clearly overrepresented in symptomatic patients compared to both exposed seronegative and the general population. Interestingly, its frequency is also increased in patients with secondary dengue hemorrhagic fever (54). Thus, higher MICB expression may positively influence DENV infection control by activating early NKG2D-mediated immune responses. A similar mechanism may be important for SARS-CoV-2 infection, and higher MICB expression may play a role in the innate immune defense against SARS-CoV-2. Conversely, variants associated with lower MICB expression, possibly implicating a diminution of NK cytotoxicity, may facilitate infection. Besides, the amino acid exchange (75-N, **Figure 3**) may also be related to ligand/receptor binding impairment or lower protein stability. In our study, rs3131639/A and MICB*004 are related to lower MICB expression (**Supplementary Figure S3**), which is confirmed by the GTEx portal (https://www.gtexportal.org). The mechanism underlying this differential expression or whether other polymorphisms in LD with rs3131639, particularly in the *MICB* promoter region, may also play a role in the expression regulation is not clear.

*MICA* alleles are also associated with susceptibility to infectious diseases (55). Here, the *MICA* variant associated with symptomatic infection is linked to higher MICA mRNA expression (**Supplementary Figure S2**). This variant is also in LD with MICA allotypes that associated with high levels of sMICA (56, 57), MICA*008 and MICA*019, both overrepresented among symptomatic individuals. High levels of soluble sMICA would have an inverse effect on NK cell activation. Other viruses are known to stimulate the release of sMICA to escape NKG2D recognition by activating endocytosis and degradation of the NKG2D receptor (58). A recent study suggested that dysregulation of the NKG2D-MICA axis could be a possible mechanism of NK cell exhaustion in SARS-CoV-2 infection, resulting in suppressive effects by excessive cytokines in the disease course. SARS-CoV-2 might escape from NKG2D recognition using a similar mechanism of elevated plasma levels of sMICA. For instance, the disintegrin and metalloproteinase17 (ADAM17) activity is upregulated upon binding of SARS-CoV to ACE2, facilitating viral entry. This might be responsible for the higher shedding of MICA after spike-ACE2 interaction during SARS-CoV-2 infection [reviewed by (59)]. Although not confirmed by protein expression analysis, we can hypothesize that the variants associated with higher MICA expression are also associated with higher sMICA levels and/or NK exhaustion, resulting in NK dysfunction in the early stages of SARS-CoV-2 infection. Indeed, decreased NK cell numbers, impaired cytotoxic activity, and a biased inflammatory phenotype have been reported in SARS-CoV-2 infection, indicating that NK cells likely integrate into the underlying immune dysregulation in COVID-19. In addition, sMICA levels were considerably higher in COVID-19 patients with severe disease (60). In this context, our findings bring additional potential mechanisms involving NK dysfunctions [reviewed by (61)], which could be confirmed by functional assays.

Innate immunity efficiency may be a critical feature between symptomatic and asymptomatic infections, and it also may facilitate the prompt elimination of the virus after exposure. Host genetics influence this feature. For instance, inborn errors of TLR3- and IRF7-dependent type I IFN immunity are associated with life-threatening COVID-19 pneumonia (24), and neutralizing autoantibodies against IFN type I were detected in 10% of patients with life-threatening COVID-19 pneumonia (62). These findings highlight the importance of the innate immunity against SARS-CoV-2. Here, we add another potential layer to this complexity—host genetics influencing NK cell activation efficiency due to differential expression capabilities of MICA and MICB.

The susceptibility haplotype formed by the *MICB* variants in **Figure 2** is more frequent in Europe (about 23%) and Africa (15%) and less frequent in Asia (9.4%). Conversely, MICA allele rs2596541/C is more frequent in East Asia (about 72%), South Asia (about 63%), and Europe (about 60%) and less frequent in Africa (about 46%). Interestingly, the *MICB* susceptibility haplotype can be detected in archaic humans, such as the Neanderthal from the Vindija Cave, and the *MICA* susceptibility allele in the Neanderthal from the Altai Mountains and one Denisovan.

### Concluding Remarks

In short, here we performed a candidate region approach to compare polymorphisms in the MHC region in symptomatic COVID-19-infected individuals and in highly exposed partners who were seronegative. We used a state-of-the-art method to call genotypes and haplotypes in the MHC. We observed little to no impact of polymorphisms in class I antigen-presentation genes, except for HLA-A allotypes carrying 144Q and 151R among asymptomatic women when the male partner is symptomatic, increasing susceptibility to symptomatic infection. We also observed an association of HLA-DRB1 alleles encoding K at residue 71 (DRB1*03:01, DRB1*04:01, and others) with susceptibility to symptomatic infections. Allele HLA-DOB*01:02 is associated with symptomatic infection mostly among women. Moreover, our results suggest that genes related to immune modulation, mainly involved in NK cell killing activation/inhibition, harbor variants potentially contributing to infection resistance. We hypothesize that individuals prone to produce higher amounts of sMICA, and low amounts of MICB, would be more susceptible to symptomatic infections. Accordingly, quantitative differences in these NK activity-related molecules could contribute to susceptibility to COVID-19, likely downregulating NK cell cytotoxic activity in infected individuals but not in resistant partners. Functional assays will provide the means to test the hypothesis of differential NK cell activity between COVID-19 symptomatic and asymptomatic exposed individuals, involving *MICA* and *MICB*.

Host genetics influencing NK cell activation due to differential expression of MICA and MICB is another layer in the complex interaction between the human genome and COVID-19 outcome. The current knowledge regarding this matter supports the link between polymorphisms and susceptibility to life-threatening COVID-19, the majority promoting moderate–higher risk. The associations described here follow the same path but with slightly higher ORs than those described in previous GWASs (4–7). For instance, the OR for MICB*004 and HLA-DOB*01:02 for symptomatic infection is 2.8 and 7.39, respectively.

Due to the likely multifactorial nature of resistance itself, the putative resistant individuals in our study could be protected by NK cell response or cytotoxic effects present due to previous endemic coronavirus with common antigenic exposure to SARS-CoV-2 (cross reaction).

## Data Availability Statement

The datasets presented in this study can be found in online repositories. The names of the repository/repositories and accession number(s) can be found in the article/**Supplementary Material**.

## Ethics Statement

This study was approved by the Committee for Ethics in Research of the Institute of Biosciences at the University of São Paulo (CAAE 34786620.2.0000.5464). The patients/participants provided their written informed consent to participate in this study.

## Author Contributions

EC, MC, MN, DM, and MZ contributed to the conceptualization. MC, LM, and MVRS contributed to data curation. EC, MN, MOS, NS, HA, AS, RP, CC, and KN contributed to the formal analysis. MZ contributed to funding acquisition. MC, MN, MOS, EC-N, and KS contributed to the investigation. EC, MN, MOS, CM-J, DM, and KN contributed to the methodology. MZ contributed to the project administration. EC, MN, and MOS contributed the software. EC, MN, MOS, CM-J, and KN contributed to visualization. EC, MC, MN, and MOS contributed to writing–original draft. NS, HA, AS, RP, CC, CM-J, DM, KN, LM, MVRS, JE, VC, RB, MH, JM, EC-N, VC, KS, MM, JK, MN, RM, MP-B, and MZ contributed to writing–review and editing. All authors contributed to the article and approved the submitted version.

## Funding

This work was supported by the São Paulo Research Foundation (FAPESP/Brazil) [grant numbers 2013/08028-1, 2014/50931-3, 2019/19998-8, and 2020/09702-1], the National Council for Scientific and Technological Development (CNPq) [grant number 465355/2014-5], and JBS S.A. [grant number 69004]. FAPESP/Brazil (Grant numbers 2013/17084-0 and 2017/19223-0) and the United States National Institutes of Health (NIH) (R01 GM075091) supported the development of the HLA and KIR pipeline and the genetic ancestry approach. This study was also supported by the Coordenação de Aperfeiçoamento de Pessoal de Nível Superior-Brasil (CAPES)-Finance Code 001 and Fleury Group (Project NP-565).

## Conflict of Interest

The authors declare that the research was conducted in the absence of any commercial or financial relationships that could be construed as a potential conflict of interest.

## Publisher’s Note

All claims expressed in this article are solely those of the authors and do not necessarily represent those of their affiliated organizations, or those of the publisher, the editors and the reviewers. Any product that may be evaluated in this article, or claim that may be made by its manufacturer, is not guaranteed or endorsed by the publisher.
