# Supplementary material for: MHC Variants Associated With Symptomatic Versus Asymptomatic SARS-CoV-2 Infection in Highly Exposed Individuals
Source: Front Immunol. 2021 Sep 28;12:742881. doi: 10.3389/fimmu.2021.742881 (PMC8506217; doi:10.3389/fimmu.2021.742881)
Supplement: Supplementary file 1 [file DataSheet_1.docx]

Supplementary Material

# Supplementary Methods

## Volunteers Recruitment and datasets

The couple's symptomatic members (COVID-19[+]) were divided into subgroups based on their COVID-19 clinical condition, using the severity scales proposed by World Health Organization  q(WHO-2019-nCoV-clinical-2020.5), and elaborated by Gandhi and collaborators[^1^](https://paperpile.com/c/l9FCha/BLBEg) . The subgroups are: (a) Asymptomatic, with no presence of symptoms [absent from this pool]; (b) mild illness, with the presence of most common symptoms such as fever, cough, dysfunction of smell (anosmia) and taste (dysgeusia) but no shortness of breath (dyspnea), and no hospitalization requirement; (c) moderate illness, with the presence of most common symptoms including dyspnea and clinical or radiographic evidence of lower respiratory tract disease but no hypoxemia (blood oxygen saturation of 94% or higher), in which hospitalization may be required; and (d) severe illness, with the presence of most common symptoms, dyspnea, hypoxemia, pulmonary impairment, and hospitalization in an intensive care unit. The infected/symptomatic member had COVID-19 confirmed by RT-PCR, and they had mild (83.1%), moderate (10.8%), or severe (6.0%) symptoms.

The couple's contactant, with no infection or any symptom despite the close contact (COVID-19[-]), presented no positive RT-PCR (for some individuals) and no positive serology test (for all individuals).

For sample collection, 6 mL of whole blood (WB) from each partner was collected after venipuncture using the BD Vacutainer tube system with ethylenediaminetripotassium (BD Catalog. 360057) for DNA extraction. DNA was automated extracted using the QIAsymphony® robot (Qiagen) with elution volumes of 200μL and following the protocols as recommended by the manufacturer. DNA quality was assessed with NanoDrop® (Thermo Fisher Scientific) and concentration with Qubit® (Thermo Fisher Scientific).

We also compared the COVID-19[+] and COVID-19[-] groups with a population-based sample from the same city [^2^](https://paperpile.com/c/l9FCha/1aEB). For this purpose, we resampled the SABE cohort (Brazilian elderly from São Paulo) using an in-house Perl script selecting 5 individuals of the same sex and similar ancestry background for each individual. Thus, these population-based samples are paired with each group by ancestry and sex. Table S1 presents the description of each dataset.

## Definition of groups


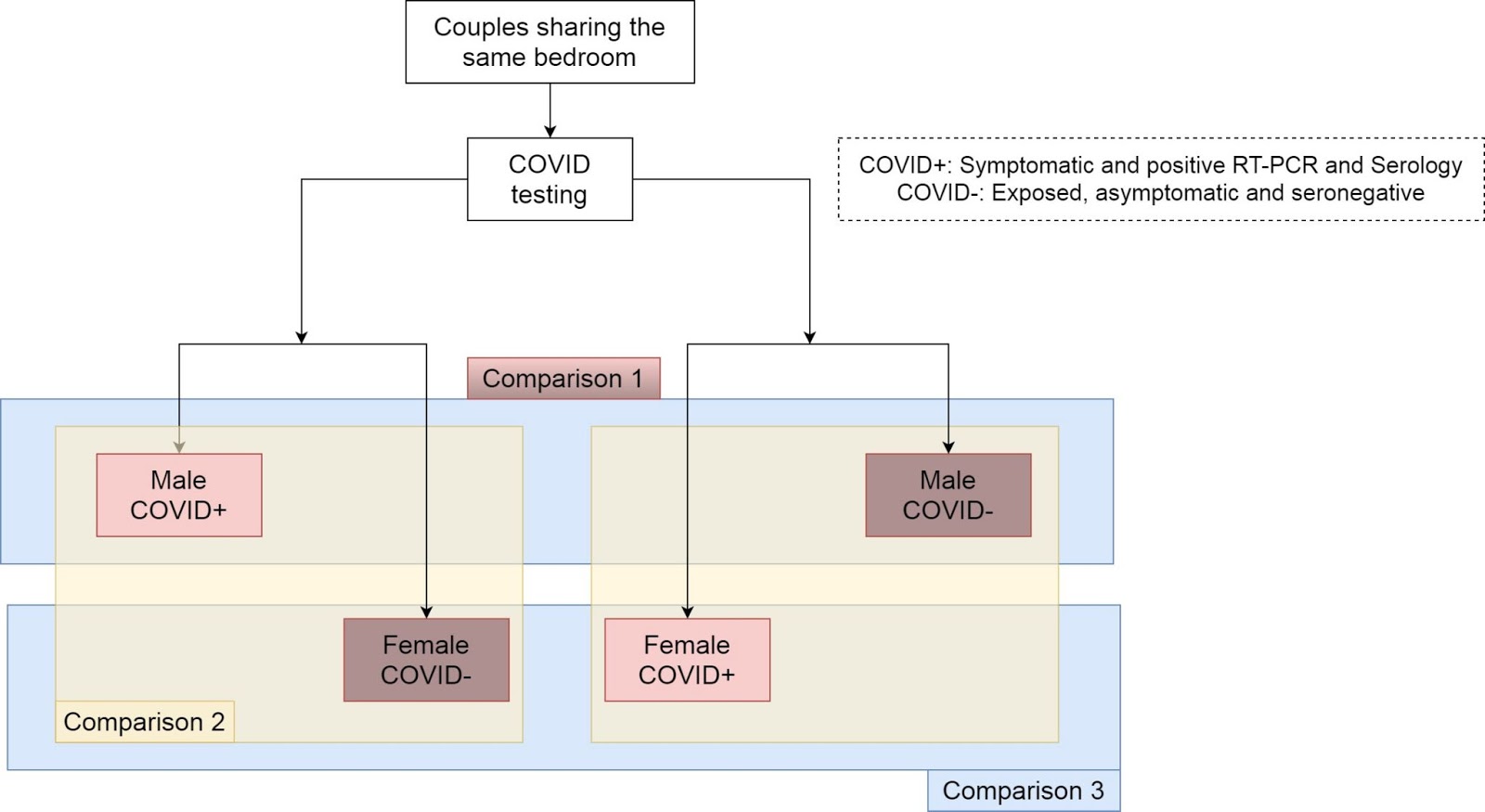


**Figure S1.** Graphical aid for groups definitions and comparisons. Comparison 1 refers to susceptibility versus resistance among all couples. Comparison 2 refers to the sex-specific direction of infection within couples. Comparison 3 refers to sex-specific analyses. All comparisons use age and genetic ancestry as covariates, and Comparison 1 includes sex as covariate.

**Table S1:** The description of the datasets and groups used to evaluate SARS-CoV-2 infection susceptibility and resistance.

| **Description** | **Size** | **Mean age** | **Sex (%Male)** | **EUR (%)** | **AFR (%)** | **EAS (%)** | **NAN (%)** |
| --- | --- | --- | --- | --- | --- | --- | --- |
| COVID-19[+] (symptomatic) | 83 | 46.8 | 61.44 | 82.77 | 8.36 | 1.09 | 7.78 |
| COVID-19[-] (asymptomatic and seronegative) | 83 | 45.9 | 34.93 | 85.98 | 6.18 | 1.90 | 5.85 |
| COVID-19[+] men | 51 | 50.2 | 100.00 | 83.35 | 8.37 | 0.37 | 7.82 |
| COVID-19[-] women | 54 | 46.3 | 0.00 | 86.25 | 5.84 | 0.00 | 5.74 |
| COVID-19[+] women | 32 | 41.4 | 0.00 | 81.17 | 7.37 | 0.41 | 7.78 |
| COVID-19[-] men | 29 | 45.0 | 100.00 | 82.41 | 6.06 | 5.27 | 6.20 |
| Population sample paired with COVID-19[+] | 415 | > 70 | 61.44 | 82.94 | 8.36 | 1.23 | 6.85 |
| Population sample paired with COVID-19[-] | 415 | > 70 | 34.93 | 85.69 | 6.38 | 2.40 | 5.47 |
| Population sample paired with COVID-19[+] men | 255 | > 70 | 100 | 84.19 | 9.38 | 0.00 | 6.32 |
| Population sample paired with COVID-19[-] women | 270 | > 70 | 0 | 82.22 | 6.13 | 0.00 | 5.56 |
| Population sample paired with COVID-19[+] women | 160 | > 70 | 0 | 84.26 | 7.59 | 0.05 | 8.01 |
| Population sample paired with COVID-19[-] men | 145 | > 70 | 100 | 81.17 | 6.42 | 6.77 | 5.55 |

EUR: Proportion of European ancestry; AFR: Proportion of African ancestry; EAS: Proportion of East Asian ancestry; NAM: Proportion of Native American ancestry.

## Exome sequencing, variant call, and variant refiniment

Reads were aligned to the human reference GRCh38 using the run-bwamem algorithm from bwa.kit 0.7.15 package (https://github.com/lh3/bwa/tree/master/bwakit). After alignment, we used Picard tools 2.18.7 (http://broadinstitute.github.io/picard) to mark duplicates and GATK 4.0.9 [^3^](https://paperpile.com/c/l9FCha/7gHCp) to perform Base Quality Score Recalibration (BQSR, BaseRecalibrator tool). Following GATK's Best Practices for germline short variant discovery [^4^](https://paperpile.com/c/l9FCha/tKLJG) and using the GATK 4.0.9 tools, we generated individual GVCFs using HaplotypeCaller (GVCF mode). We then combined the GVCFs of all individuals with CombineGVCFs to jointly call variants using GenotypeGVCFs and perform Variant Quality Score Recalibration (VQSR, VariantRecalibrator). The mean depth of coverage for the 172 individuals was 95X, ranging from 75 to 150X.

We checked the biological sex of individuals against informed sex using the coverage ratio between the X and Y chromosomes. We used PC-Relate implemented in the GENESIS R package to check for relatives or contamination in the sample [^5^](https://paperpile.com/c/l9FCha/DmvEP). No individual failed sex or kinship checks.

## Ancestry assessment

We used our previously developed CEGH-Filter to evaluate the quality of called variants and genotypes [^2^](https://paperpile.com/c/l9FCha/1aEB). For analysis, we removed GATK non-PASS, CEGH, non-vSR, and multiallelic variants.

Ancestry was inferred in ADMIXTURE v1.3[^6^](https://paperpile.com/c/l9FCha/yLFdu), based on likelihood models and the information about allele frequencies of the different parental populations. As parental populations, we used samples from the 1000 Genomes project [^7^](https://paperpile.com/c/l9FCha/wBMhr) and HGDP-CEPH [^8^](https://paperpile.com/c/l9FCha/HD6dZ) totalizing 602 African, 624 European,  630 East Asian, and 118 Native American, with more than 95% of ancestry inferred for that ethnic group, according to the analysis unsupervised (K = 4) in ADMIXTURE. Ancestry inferences were performed with supervised analysis (K = 4) after applying linkage disequilibrium filters (*r^2^* = 0.1) within a sliding window of 50Kb and a shift step of 10Kb, totaling 50,995 SNPs. Lastly, we retrieved the proportion of each ancestry (European, African, Native American, and East Asian) for each sample, which was used as covariates in subsequent analyses, and to select the population-based samples paired to each group.

## MHC mapping optimization, genotyping, and haplotyping

The workflow used in this study is illustrated in Figure S2. First, we aligned reads to the human reference GRCh38 using the run-bwamem algorithm from bwa.kit 0.7.15 package (<https://github.com/lh3/bwa/tree/master/bwakit>) as described earlier. The SAM file produced by using BWA was converted to a BAM file, sorted, and indexed using Samtools [^9^](https://paperpile.com/c/l9FCha/4We51). This BAM file is the input for the MHC alignment optimization by using hla-mapper.

We used hla-mapper version 4.1.0 ([www.castelli-lab.net/apps/hla-mapper](http://www.castelli-lab.net/apps/hla-mapper)) to optimize alignments in the MHC region [^10^](https://paperpile.com/c/l9FCha/T5vDk), using a similar approach as previous manuscripts addressing HLA in Brazil [^2,11,12^](https://paperpile.com/c/l9FCha/1aEB+5vUS+AWzh). The MHC region is prone to alignment and genotyping errors [^10,13^](https://paperpile.com/c/l9FCha/T5vDk+xI7w7), and the hla-mapper algorithm minimizes these errors allowing a more accurate genotyping and haplotyping procedure. The end product of such optimization is a BAM file for each sample. The analysis workflow is described here: <https://github.com/erickcastelli/HLA_genotyping>.

For variant calling, we used GATK HaplotypeCaller in the GVCF mode [^4^](https://paperpile.com/c/l9FCha/tKLJG). After converting the GVCF files to VCF by using GATK GenotypeGVCFs, we recoded the VCF file using vcftools to correct minor encoding errors [^14^](https://paperpile.com/c/l9FCha/XCCSJ). After, we proceeded with a variant refinement step by first using vcfx ([www.castelli-lab.net/apps/vcfx](http://www.castelli-lab.net/apps/vcfx)) algorithm checkpl (genotype likelihood = 0.95), which introduces missing alleles in genotypes with low likelihood (for example, an homozygous genotype in a region with only two reads, or an heterozygous genotype in which 90% of all reads point to the same nucleotide), and second with algorithm vcfx evidence (default parameters), which annotates each variant with quality control parameters (number of heterozygous sites with even distribution of reads per allele, number of homozygous genotypes for alternative alleles, and others). Then, we used vcfx filter to recover only variants annotated as PASS or WARN in the previous steps. The final step for variant refinent is the removal of alternative alleles that are no longer present in the dataset, recoding the VCF file by using *Bamtools view* --trim-alt-alleles --min-ac 1 [^15^](https://paperpile.com/c/l9FCha/ESlUZ). After, we filtered variants that coincided with the regions that are captured by the IDT xgen-V1, with a tolerance of 100 nucleotides upstream and downstream each region. We also filtered the variants that coincide with the regions optimized by hla-mapper (Figure 1). The end-product of these procedures is an unphased VCF file (Figure S2) that is used for the association analysis described later, and also used as input for the haplotyping procedure.

To call haplotypes, we combined read-aware phasing and probabilistic models. First, we converted multi-allelic variants into bi-allelic ones using *Bamtools norm*. Then, we used GATK ReadBackedPhasing (from GATK 3.8) to infer the physical phasing between closely related variants. Similar results can be obtained with WhatsHap [^16^](https://paperpile.com/c/l9FCha/JKYcQ). This step can be parallelized using a Perl script to speed up the process, since neither the ReadBackedPhasing nor WhatsHap are multithreaded. This script is available at <https://github.com/erickcastelli/phasex>. The VCF file containing the phased data is the input for the haplotyping procedure using a local program named phasex (<https://github.com/erickcastelli/phasex>).

Phasex is a program that automates multiple haplotyping runs by using Shapeit 4 [^17^](https://paperpile.com/c/l9FCha/E3Igl) and the data obtained by ReadBackedPhasing or WhatsHap. It performs many independent runs, comparing the results afterwards, fixing haplotypes for a sample in which at least 95% of the runs indicate the same results. Then, this step is repeated many times until the number of samples with the same haplotype at least 95% of the runs do not increase any further. The end product of this procedure is phased bi-allelic VCF, which is then converted to multi-allelic variants using *bamtools norm*. It should be mentioned that we have evaluated each MHC gene separately by retrieving the variants corresponding to each locus, and running phasex for each gene.The missing alleles introduced by vcfx are imputed by Shapeit 4.


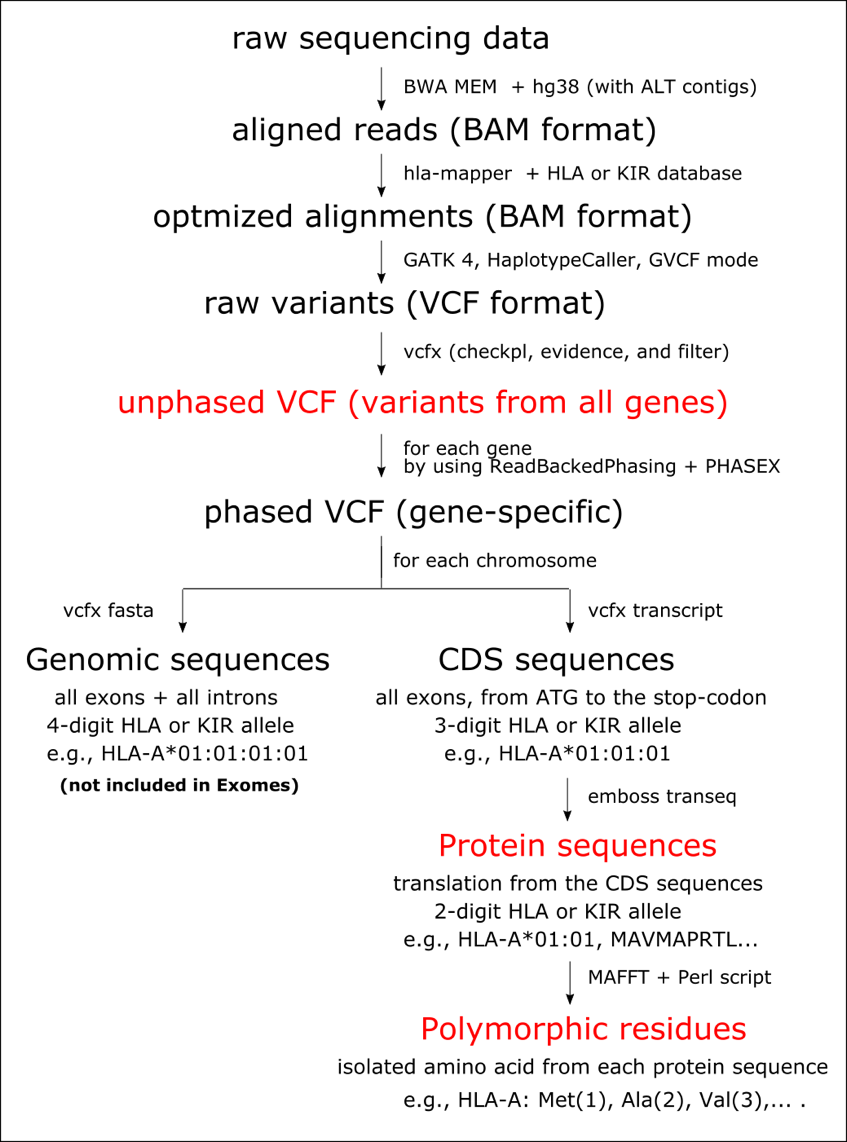


**Figure S2**: The workflow used in this study, from the raw sequencing data to complete sequences for genes from the MHC. We used the datasets marked in red for the SARs-CoV-2 association analyses.

## Getting the name of HLA alleles

Using a Perl script to automate the process, available at (<https://github.com/erickcastelli/HLA_genotyping>), we first exported the phased VCF to complete sequences (only exons) of each gene. To do that, we used vcfx transcript, indicating chromosome 6 as a reference, the phased VCF, and a BED file with coordinates of each exon (starting from the first translated ATG). This procedure produces two sequences per individual, one for each chromosome. Then, we detected each different sequence, comparing them with the ones available in the IPD-IMGT/HLA database [^18^](https://paperpile.com/c/l9FCha/qSNPY). When the sequence we detected was found in the database, we updated its name accordingly. When it was a new sequence, we named the sequence as a new one (Figure S2).

After, we translated the sequence of each chromosome using emboss transeq, generating a fasta file with the proteins encoded by each chromosome of each individual, considering the canonical transcript as reference. Then, we detected each different sequence, also naming them following the ones in the IPD-IMGT/HLA database, or identifying them as new sequences. All these steps are automated by the Perl script ((<https://github.com/erickcastelli/HLA_genotyping>)), which generates fasta files as described above, and a final database indicating the alleles of each individual considering the exonic sequence and the protein sequence (Figure S2).

## Statistical analyses for the association study

First, we tested for the association between bi-allelic and multi-allelic variants and two phenotypes: COVID-19[-] and COVID-19[+]. We compared these groups (comparison 1), and also stratifying them by sex (comparisons 2 and 3). We also compared COVID-19[-] and COVID-19[+] groups with their paired population-based samples. Because there are many multi-allelic variants in the MHC, to perform the logistic regression, we used a local Perl script to convert the unphased VCF file (Figure S2) to a plink-like table in which each allele of a variant is considered an independent marker. Genotypes carrying missing alleles are disregarded by registering "NA" to the presence of this marker. Then, we used R to fit a regression model for each marker, controlling for sex, age, and genetic ancestry [we have not controlled for sex when dealing with the sex-stratified groups]. We filtered all markers that presented a P-value lower than 0.5% (< 0.005), summarizing these markers and their frequencies in Figure 2.

After translating the exonic sequences to proteins (Figure S2), we explored whether specific protein sequences within genes from the MHC are associated with the phenotypes. We used a similar Perl script to create a plink-like table that considers each different allotype as an independent marker, and indicates the number of copies of these markers for each sample. Likewise performed for SNPs, we tested the frequency of each different protein sequence (the allotypes) among groups by using logistic regression and R, also controlling for sex, age, ancestry, also  considering a threshold o P < 0.005 (Figure 3).

Because more than one allele in multi-allelic variants may encode the same amino acid, and because multiple full-length proteins may present the same amino acid in one specific position, we also tested the frequency of every amino acid residue. First, for each locus, we aligned the complete protein sequences of every individual (two per individual) by using MAFFT (Figure S2). Afterwards, we used a Perl script that created a plink-like table with every allele (different amino acid) in every position as an independent marker, and the number of copies of this marker in each sample. We also fitted a logistic regression by using R, also controlling for sex, age, ancestry, as performed previously (Figure 3).

## Expression analysis in the GEUVADIS dataset

For some variants associated with SARS-CoV-2 infection susceptibility, we investigated whether these variants influence the expression of their respective genes. We used the GEUVADIS dataset of RNA-seq data from 460 samples from the 1000 Genomes project [^19^](https://paperpile.com/c/l9FCha/G6vTT) to evaluate the expression levels. First, we genotyped all samples in the new 1000 Genomes release of high-coverage sequencing [^20^](https://paperpile.com/c/l9FCha/7PIWL), by applying hla-mapper to optimize the BAM files available for download and applying the same pipeline to call and refine genotypes (Figure S2).

Second, we aligned the raw sequencing data from GEUVADIS using STAR [^21^](https://paperpile.com/c/l9FCha/Cugsi) and the human genome draft as reference (version hg38 without ALT contigs), considering all known transcripts annotated for the hg38 genome. Then, we applied a beta version of the hla-mapper suitable for RNA-seq data (on development), that optimizes the alignment of the same genes as for the DNA version. With these optimized alignments, we used subread featureCounts [^22^](https://paperpile.com/c/l9FCha/0o4oA) to count the number of reads aligned in each gene. We used the total number of aligned reads and the length of each gene as indicated by subread to calculate TPM for each locus.

Third, for each tested variant, by using a local Perl script, we tracked the TPM values for individuals carrying different genotypes, producing a text file composed of the sample name, its genotype, and the TPM value for a specific locus. By using R, we compared the distribution of TPM values among individuals carrying different genotypes (using wilcoxon or t-test), plotting this distribution in boxplots.

## Evaluation of the susceptibility markers in archaic humans

We have downloaded the aligned BAM files from two Neanderthals from the Altai Mountains [^23^](https://paperpile.com/c/l9FCha/EHN6D) and the Vindija Cave in Croatia [^24^](https://paperpile.com/c/l9FCha/211L7), and one Denisovan [^25^](https://paperpile.com/c/l9FCha/8aqKn). Then, we applied hla-mapper to optimize the alignment in the MHC region. By using Integrative Genome Viewer (https://software.broadinstitute.org/software/igv/), we manually evaluated each associated position described in Figure 2 (upper panel).

# Supplementary Results

**Table S2**: The frequency of *HLA-A* and *HLA-B* alleles grouped as supertypes

| **Supertype** | **Gene** | **COVID-19[-]** | **COVID-19[+]** | **P-value** |
| --- | --- | --- | --- | --- |
| A01 | HLA-A | 0.1928 | 0.2410 | 0.0678 |
| A01A03 | HLA-A | 0.0241 | 0.0060 | 0.2191 |
| A01A24 | HLA-A | 0.0663 | 0.0482 | 0.4405 |
| A02 | HLA-A | 0.2530 | 0.2289 | 0.5580 |
| A03 | HLA-A | 0.1687 | 0.2410 | 0.0378 |
| A24 | HLA-A | 0.1988 | 0.1506 | 0.0885 |
| B07 | HLA-B | 0.2771 | 0.3554 | 0.0969 |
| B08 | HLA-B | 0.0723 | 0.0723 | 0.5982 |
| B27 | HLA-B | 0.1325 | 0.1446 | 0.9241 |
| B44 | HLA-B | 0.3313 | 0.2530 | 0.0655 |
| B58 | HLA-B | 0.0663 | 0.0602 | 0.6872 |
| B62 | HLA-B | 0.0241 | 0.0542 | 0.3526 |
| **Supertype** | **Gene** | **COVID-19[-] women** | **COVID-19[+] men** | **P-value** |
| A01 | HLA-A | 0.2100 | 0.2400 | 0.3360 |
| A01A03 | HLA-A | 0.0300 | 0.0100 | 0.3730 |
| A01A24 | HLA-A | 0.0800 | 0.0300 | 0.0512 |
| A02 | HLA-A | 0.2400 | 0.2400 | 0.9919 |
| A03 | HLA-A | 0.1400 | 0.2000 | 0.4500 |
| A24 | HLA-A | 0.1900 | 0.1900 | 0.7980 |
| B07 | HLA-B | 0.2900 | 0.3400 | 0.1510 |
| B08 | HLA-B | 0.0600 | 0.0900 | 0.5830 |
| B27 | HLA-B | 0.1400 | 0.1500 | 0.6010 |
| B44 | HLA-B | 0.3200 | 0.2500 | 0.1080 |
| B58 | HLA-B | 0.0500 | 0.0500 | 0.8040 |
| B62 | HLA-B | 0.0200 | 0.0500 | 0.4090 |
| **Supertype** | **Gene** | **COVID-19[-] men** | **COVID-19[+] women** | **P-value** |
| A01 | HLA-A | 0.1786 | 0.2321 | 0.2255 |
| A01A03 | HLA-A | 0.0179 | 0.0000 | 0.4237 |
| A01A24 | HLA-A | 0.0536 | 0.0714 | 0.9940 |
| A02 | HLA-A | 0.2679 | 0.2143 | 0.8855 |
| A03 | HLA-A | 0.1964 | 0.3214 | 0.1176 |
| A24 | HLA-A | 0.1964 | 0.0714 | 0.0724 |
| B07 | HLA-B | 0.2321 | 0.3393 | 0.4679 |
| B08 | HLA-B | 0.1071 | 0.0536 | 0.1866 |
| B27 | HLA-B | 0.1429 | 0.1071 | 0.7257 |
| B44 | HLA-B | 0.3214 | 0.3036 | 0.9013 |
| B58 | HLA-B | 0.0893 | 0.0714 | 0.7316 |
| B62 | HLA-B | 0.0357 | 0.0714 | 0.4221 |


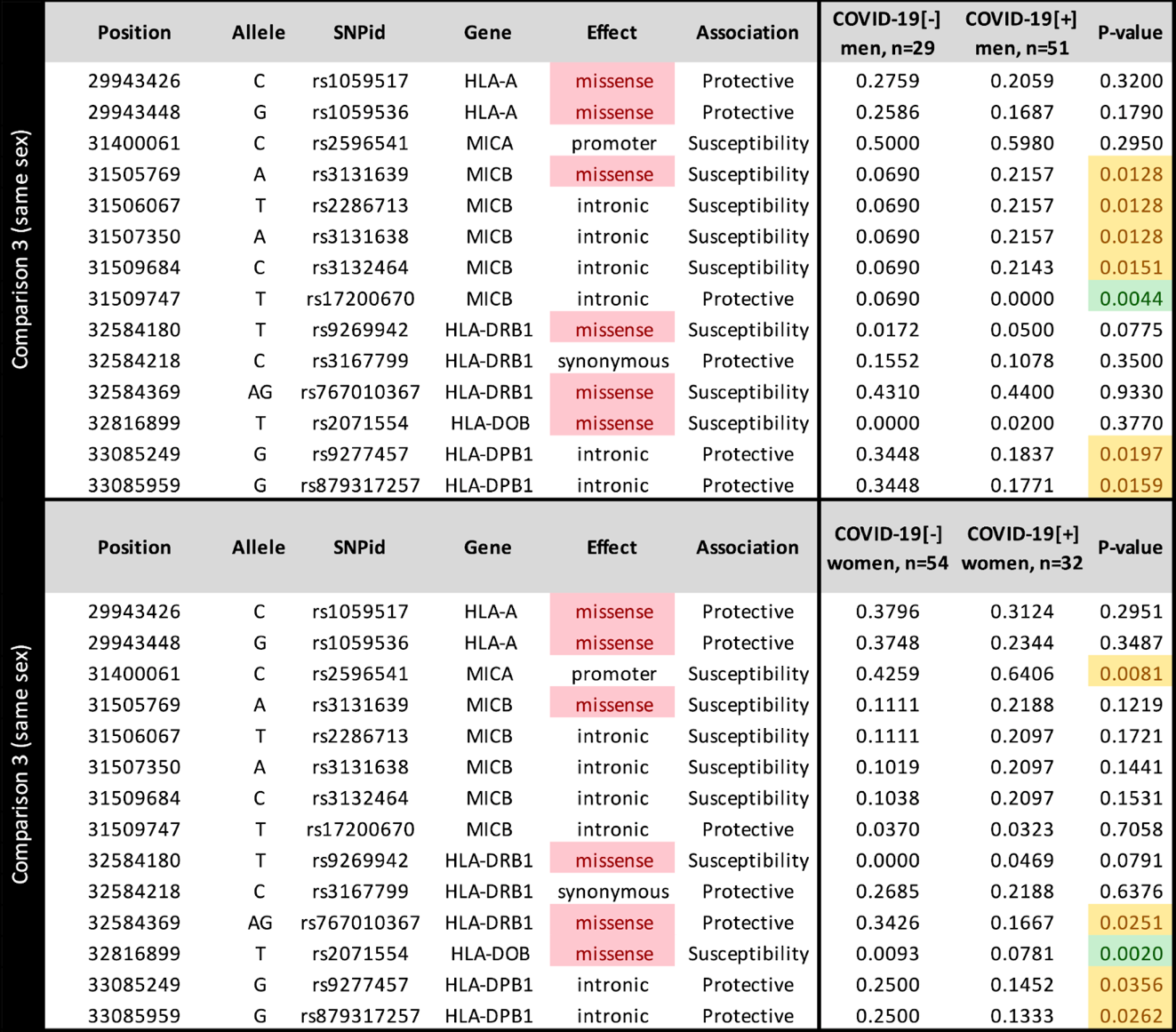


**Figure S3:** The frequency of each candidate variant at the Major Histocompatibility Complex (MHC) associated with susceptibility to SARS-CoV-2 symptomatic infection or with asymptomatic and seronegative after exposure, in individuals with the same sex. COVID-19[+]: Patients with symptomatic COVID-19. COVID-19[-]: Individuals sharing the same bed with symptomatic patients (exposed individuals) and are asymptomatic and seronegative.  P-value: Logistic regression comparing COVID-19[+] and COVID-19[-] individuals, controlling for age, and genetic ancestry. In green, P-values < 0.005; In yellow, P-value between 0.005 and 0.05.


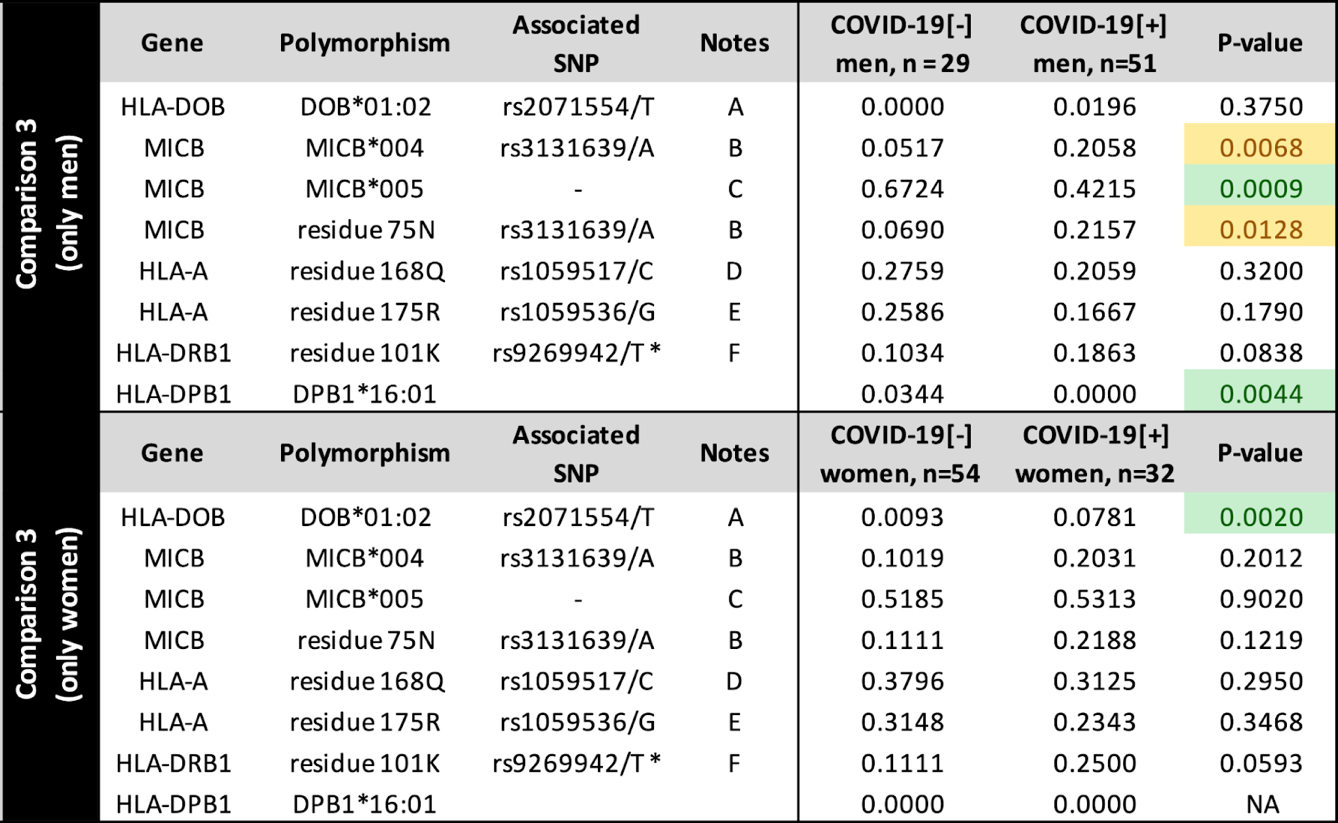


**Figure S4:** The frequency of each candidate allotype or amino acid residue at the Major Histocompatibility Complex (MHC) associated with susceptibility to SARS-CoV-2 symptomatic infection or with asymptomatic and seronegative after exposure, in individuals with the same sex. COVID-19[+]: Patients with symptomatic COVID-19. COVID-19[-]: Individuals sharing the same bed with symptomatic patients (exposed individuals) and are asymptomatic and seronegative.  P-value: Logistic regression comparing COVID-19[+] and COVID-19[-] individuals, controlling for age, and genetic ancestry. In green, P-values < 0.005; In yellow, P-value between 0.005 and 0.05.

## Potential mechanisms underlying the associations

### MICA, variant rs2596541

*MICA* is also induced by stress and is a ligand for the NKG2D. We detected an association with rs2596541, located more than 3.5 Kb upstream the *MICA* first translated ATG. This variant is an eQTLs for *MICA* expression in the GTEx portal, with the susceptibility allele (rs2596541/C) associated with higher MICA mRNA expression levels in many tissues, including lung. This variant lay on a Candidate cis-Regulatory Element (cCRE), EH38E3459116, with a proximal enhancer-like signiture, and a CpG island of 440 nucleotides [^26^](https://paperpile.com/c/l9FCha/HIRW0). The association between rs2596541/C and higher *MICA* mRNA expression levels was confirmed in the GEUVADIS dataset, Figure S2.

By evaluating 5,350 samples from worldwide populations, including 1,171 Brazilians from the SABE dataset [^2^](https://paperpile.com/c/l9FCha/1aEB) (data not shown), we detected that rs2596541/C present a strong linkage disequilibrium with some MICA allotypes: MICA*008, MICA*010, MICA*018, MICA*019, MICA*027, and others. Of those, MICA*008 and MICA*019 have been reported as high-expressing alleles, particularly the soluble isoform [^27,28^](https://paperpile.com/c/l9FCha/GKNW+RT7z). MICA*008 and MICA*019 are highly similar in the extracellular domains, and they differ in the C-terminal because MICA*008 presents a premature stop codon [^29,30^](https://paperpile.com/c/l9FCha/nlUJ+f0eQ). While MICA*008 is released via the exosomal pathway, MICA*019 is released via proteolytic cleavage [^31^](https://paperpile.com/c/l9FCha/MXAQ). Both these alleles are overrepresented among symptomatic patients and present borderline P-values when comparing symptomatic and asymptomatic seronegative individuals (P = 0.0434 for MICA*008, and P = 0.0169 for MICA*019). Thus, it is plausible that individuals carrying rs2596541/C are prone to an over-expression of MICA by two mechanisms: (a) higher mRNA expression due to differential bind of transcription factors, and (b) higher chance of carrying MICA allotypes that produce more soluble MICA. This over-expression of soluble MICA may lead to the down-regulation of the NK cell cytotoxic activity in symptomatic individuals but not in asymptomatic one.

 
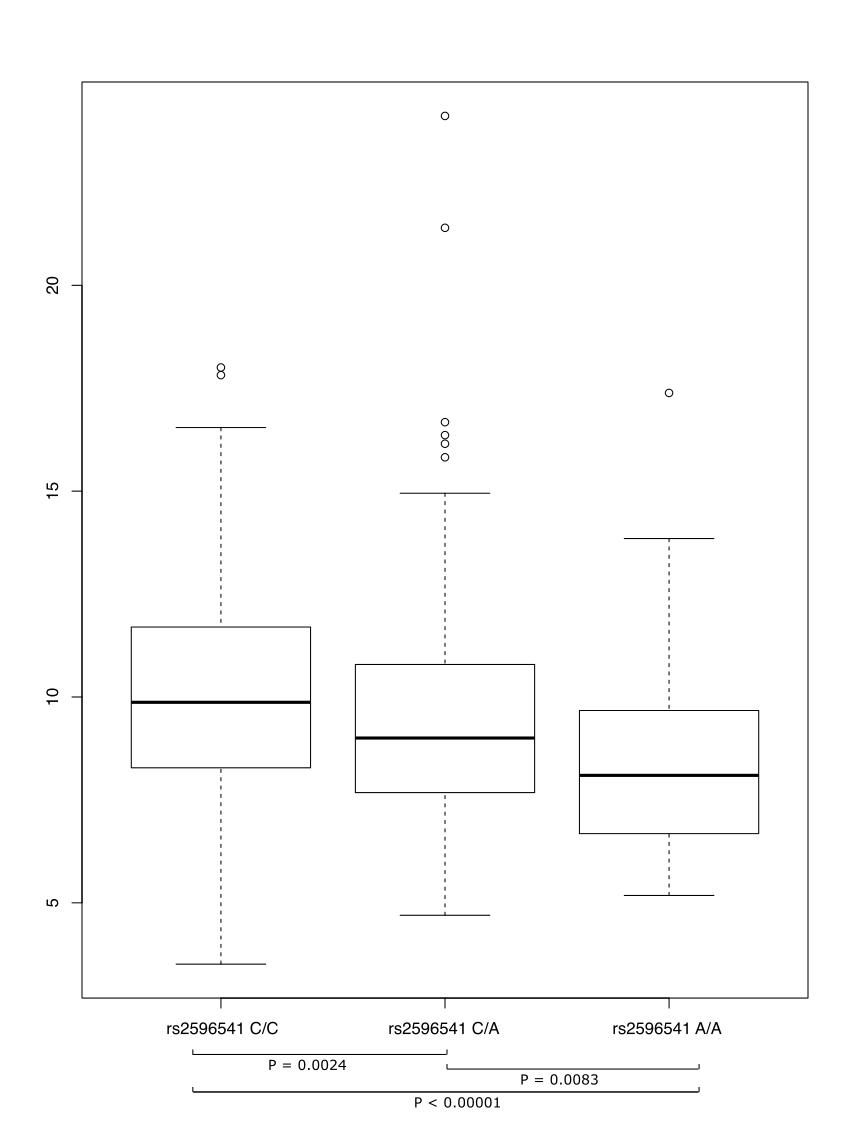


**Figure S5**: MICA mRNA expression profile for individuals from the GEUVADIS cohort presenting different genotypes for rs2596541.

### MICB*004 and rs3131639

*MICB* encodes a stress-induced molecule (e.g., during infection diseases) that is a ligand for the NKG2D type II receptor, modulating cytotoxicity of T and NK cells. rs3131639 reference allele (Adenine) encodes Asn (neutral, polar) while the alternative allele Guanine encodes Asp (acid, charged). rs3131639/A defines the allele MICB*004:01. Allele rs3131639/A is associated with lower *MICB* mRNA expression according to the GTEx portal. We evaluated the expression profile of 438 individuals from the GEUVADIS dataset [^19^](https://paperpile.com/c/l9FCha/G6vTT), and rs3131639/A was also associated with lower levels of mRNA (Figure S3). It is not clear the mechanism underlying these expression levels since none of these SNPs seem to coincide with regulatory elements [^26^](https://paperpile.com/c/l9FCha/HIRW0). Thus, we can hypothesize that individuals carrying MICB*004/rs3131639A are prone to express less MICB than non-carriers, thus activating less NK cells via NKG2D, facilitating infection.


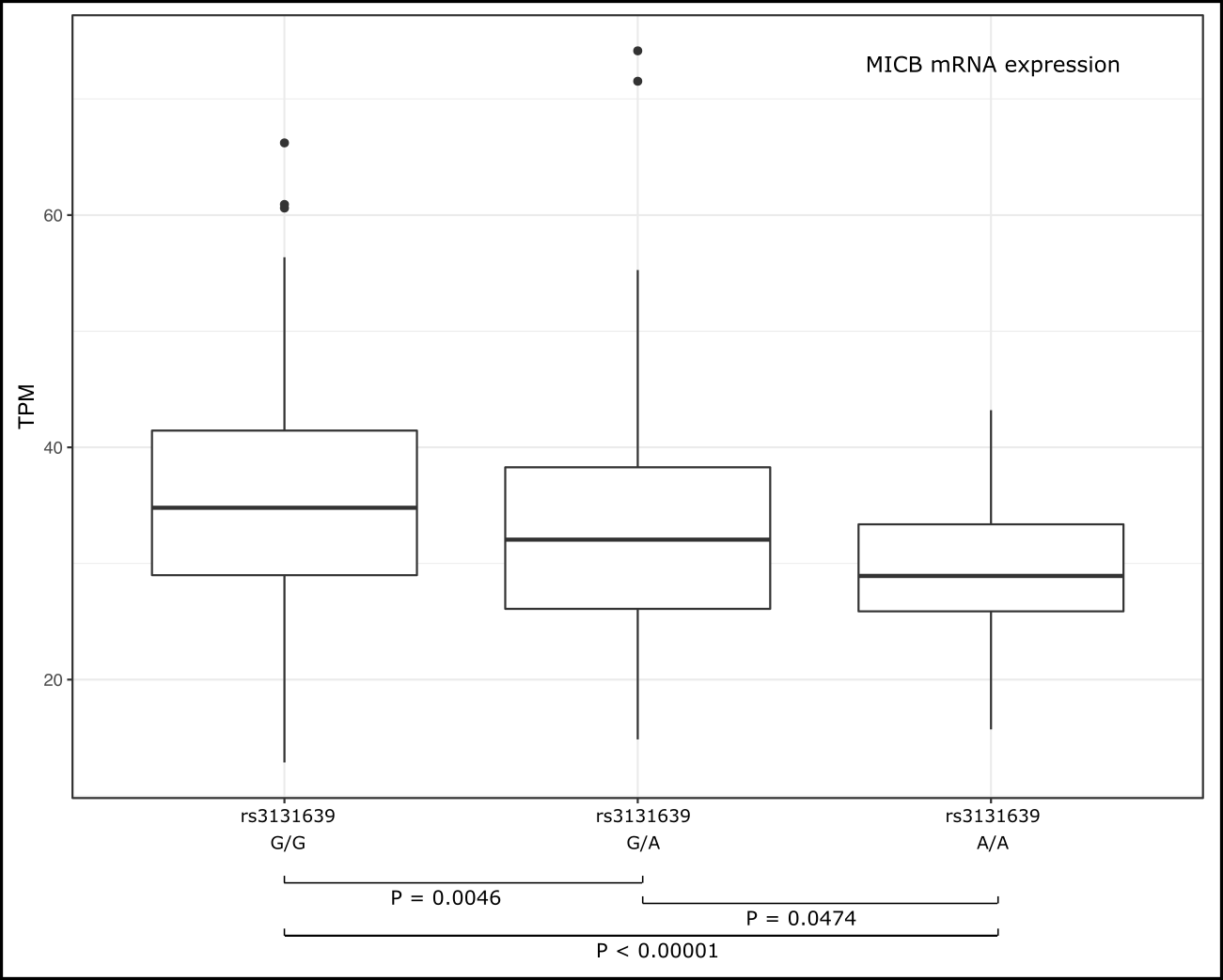


Figure S6: MICB mRNA expression profile for individuals from the GEUVADIS cohort presenting different genotypes for rs3131639.

### DOB*01:02 and rs2071554

HLA-DO is a heterodimer formed by HLA-DOA and HLA-DOB, and it is usually located in intracellular vesicles anchored to the membrane. Variant rs2071554/T is a missense variation that results in an arginine to glutamine substitution in the HLA-DOB signal peptide, at position 18 (R18Q). Polyphen2 analysis [^32^](https://paperpile.com/c/l9FCha/Kul91) predicted this amino acid exchange as possible damaging to the protein function (Polyphen2: 0.897, sensitivity: 0.82, specificity: 0.94). Similarly, SIFT [^33^](https://paperpile.com/c/l9FCha/pbxIV) predicted this exchange to be deleterious (SIFT score: 0.016). However, the impact of this exchange in protein function is not clear. Besides the modification in the signal peptide, it is unclear if this variant influences HLA-DOB cellular localization and expression. While SignalP-5 [^34^](https://paperpile.com/c/l9FCha/xvjIE) predicts that both signal peptides (the most frequent one and the one carrying 18Q due to rs2071554) as secretory signal peptides, PrediSi (<http://www.predisi.de>) predicts only the mutated one as a secretion peptide. Therefore, it is possible that cellular localization is influenced by this mutation. According to GTEx and our analysis of the GEUVADIS dataset (*P* = 0.0643), this variant does not influence HLA-DOB expression levels. However, the region encompassing rs2071554 presents a cCRE with a promoter-like signature [^26^](https://paperpile.com/c/l9FCha/HIRW0).

# References

1. Gandhi, R. T., Lynch, J. B. & Del Rio, C. Mild or Moderate Covid-19. N. Engl. J. Med. 383, 1757–1766 (2020).

2. Naslavsky, M. S. et al. Whole-genome sequencing of 1,171 elderly admixed individuals from the largest Latin American metropolis (São Paulo, Brazil). Cold Spring Harbor Laboratory 2020.09.15.298026 (2020) doi:10.1101/2020.09.15.298026.

3. McKenna, A. et al. The Genome Analysis Toolkit: a MapReduce framework for analyzing next-generation DNA sequencing data. Genome Res. 20, 1297–1303 (2010).

4. Van der Auwera, G. A. et al. From FastQ data to high confidence variant calls: the Genome Analysis Toolkit best practices pipeline. Curr. Protoc. Bioinformatics 43, 11.10.1–11.10.33 (2013).

5. Conomos, M. P., Miller, M. B. & Thornton, T. A. Robust inference of population structure for ancestry prediction and correction of stratification in the presence of relatedness. Genet. Epidemiol. 39, 276–293 (2015).

6. Alexander, D. H., Novembre, J. & Lange, K. Fast model-based estimation of ancestry in unrelated individuals. Genome Res. 19, 1655–1664 (2009).

7. 1000 Genomes Project Consortium et al. A global reference for human genetic variation. Nature 526, 68–74 (2015).

8. Clarke, L. et al. The international Genome sample resource (IGSR): A worldwide collection of genome variation incorporating the 1000 Genomes Project data. Nucleic Acids Res. 45, D854–D859 (2017).

9. Li, H. et al. The Sequence Alignment/Map format and SAMtools. Bioinformatics 25, 2078–2079 (2009).

10. Castelli, E. C., Paz, M. A., Souza, A. S., Ramalho, J. & Mendes-Junior, C. T. Hla-mapper: An application to optimize the mapping of HLA sequences produced by massively parallel sequencing procedures. Hum. Immunol. 79, 678–684 (2018).

11. Souza, A. S. et al. Hla-C genetic diversity and evolutionary insights in two samples from Brazil and Benin. Hladnikia 96, 468–486 (2020).

12. Lima, T. H. A. et al. HLA-A promoter, coding, and 3’UTR sequences in a Brazilian cohort, and their evolutionary aspects. Hladnikia 93, 65–79 (2019).

13. Brandt, D. Y. C. et al. Mapping Bias Overestimates Reference Allele Frequencies at the HLA Genes in the 1000 Genomes Project Phase I Data. G3 5, 931–941 (2015).

14. Danecek, P. et al. The variant call format and VCFtools. Bioinformatics 27, 2156–2158 (2011).

15. Barnett, D. W., Garrison, E. K., Quinlan, A. R., Strömberg, M. P. & Marth, G. T. BamTools: a C++ API and toolkit for analyzing and managing BAM files. Bioinformatics 27, 1691–1692 (2011).

16. Patterson, M. et al. WhatsHap: Weighted Haplotype Assembly for Future-Generation Sequencing Reads. J. Comput. Biol. 22, 498–509 (2015).

17. Delaneau, O., Zagury, J.-F., Robinson, M. R., Marchini, J. L. & Dermitzakis, E. T. Accurate, scalable and integrative haplotype estimation. Nature Communications vol. 10 (2019).

18. Robinson, J. et al. The IPD and IMGT/HLA database: allele variant databases. Nucleic Acids Res. 43, D423–31 (2015).

19. Lappalainen, T. et al. Transcriptome and genome sequencing uncovers functional variation in humans. Nature 501, 506–511 (2013).

20. Byrska-Bishop, M. et al. High coverage whole genome sequencing of the expanded 1000 Genomes Project cohort including 602 trios. bioRxiv (2021) doi:10.1101/2021.02.06.430068.

21. Dobin, A. et al. STAR: ultrafast universal RNA-seq aligner. Bioinformatics 29, 15–21 (2013).

22. Liao, Y., Smyth, G. K. & Shi, W. featureCounts: an efficient general purpose program for assigning sequence reads to genomic features. Bioinformatics 30, 923–930 (2014).

23. Prüfer, K. et al. The complete genome sequence of a Neanderthal from the Altai Mountains. Nature 505, 43–49 (2014).

24. Prüfer, K. et al. A high-coverage Neandertal genome from Vindija Cave in Croatia. Science 358, 655–658 (2017).

25. Meyer, M. et al. A high-coverage genome sequence from an archaic Denisovan individual. Science 338, 222–226 (2012).

26. Davis, C. A. et al. The Encyclopedia of DNA elements (ENCODE): data portal update. Nucleic Acids Res. 46, D794–D801 (2018).

27. Wang, C.-M. et al. Allele and Soluble MICA as Biomarkers for Ankylosing Spondylitis in Taiwanese. J Pers Med 11, (2021).

28. Shi, C. et al. Allele Specific Expression of MICA Variants in Human Fibroblasts Suggests a Pathogenic Mechanism. Open Rheumatol. J. 9, 60–64 (2015).

29. Ashiru, O. et al. A GPI anchor explains the unique biological features of the common NKG2D-ligand allele MICA*008. Biochem. J 454, 295–302 (2013).

30. Zou, Y. et al. Genomic characterization of MICA gene using multiple next generation sequencing platforms: A validation study. Hladnikia 96, 430–444 (2020).

31. Ashiru, O. et al. Natural killer cell cytotoxicity is suppressed by exposure to the human NKG2D ligand MICA*008 that is shed by tumor cells in exosomes. Cancer Res. 70, 481–489 (2010).

32. Adzhubei, I. A. et al. A method and server for predicting damaging missense mutations. Nat. Methods 7, 248–249 (2010).

33. Vaser, R., Adusumalli, S., Leng, S. N., Sikic, M. & Ng, P. C. SIFT missense predictions for genomes. Nat. Protoc. 11, 1–9 (2016).

34. Almagro Armenteros, J. J. et al. SignalP 5.0 improves signal peptide predictions using deep neural networks. Nat. Biotechnol. 37, 420–423 (2019).
